# Epicardial adipose tissue: a potential biomarker and therapeutic target in patients with paradoxical low-gradient aortic stenosis and preserved ejection fraction

**DOI:** 10.1097/MS9.0000000000004035

**Published:** 2025-10-06

**Authors:** Qamar Abbas, Muhammad Ali Johar, Maliha Khalid, Muhammad Talha, Aminath Waafira

**Affiliations:** a Department of Medicine, King Edward Medical University, Lahore, Pakistan; b Department of Medicine, Jinnah Sindh Medical University, Karachi, Pakistan; c Department of Medicine, King Edward Medical University, Lahore, Pakistan; d School of Medicine, The Maldives National University, Malé, Maldives

**Keywords:** aortic stenosis, cardiac remodeling, epicardial adipose tissue, low-gradient AS

## Abstract

Paradoxical low-flow, low-gradient (LFLG) aortic stenosis (AS) with preserved ejection fraction (ASpEF) is a severe AS subtype associated with worse prognosis and increased mortality compared with high-gradient AS. Epicardial adipose tissue (EAT), a metabolically active visceral fat depot surrounding the myocardium, has emerged as a potential biomarker and therapeutic target in this population. EAT influences myocardial and valvular pathology through mechanical compression, metabolic alterations, and secretion of inflammatory mediators such as IL-6, MCP-1, and TNF-α, contributing to left ventricular remodeling and aortic valve degeneration. Recent metabolomic studies demonstrate significant associations between EAT thickness and both positively and negatively correlated metabolites, underscoring its complex role in disease progression. Despite its promise, challenges remain due to limited understanding of EAT–myocardium interactions, variable patient presentation, and confounding factors such as insulin resistance and obesity. Further research is warranted to clarify EAT’s pathophysiological role, prognostic value, and therapeutic potential in managing paradoxical LFLG ASpEF.

*To the Editor,*

Paradoxical low-flow, low-gradient aortic stenosis (AS) is a severe AS subtype with a small valve area (≤1.0 cm^2^), low mean gradient (<40 mmHg), and reduced stroke volume index (≤35 mL/m^2^) despite preserved ejection fraction (≥50%). Low stroke volume arises primarily from concentric left ventricular (LV) hypertrophy, causing a small cavity size and impaired diastolic filling often due to long-standing hypertension and may be exacerbated by atrial fibrillation, valvular regurgitation or stenosis, right ventricular dysfunction, or wild-type transthyretin amyloidosis. It is common in elderly, hypertensive, and female patients and has a generally worse prognosis and higher mortality compared with patients with high-gradient severe AS^[1]^.

Epicardial adipose tissue (EAT) is a visceral fat depot between the myocardium and visceral pericardium. It shares blood supply with the myocardium and enables paracrine interaction as it covers 80% of the heart’s surface and accounts for ~14.7% of heart weight. It plays metabolic, mechanical, and endocrine roles, including thermogenesis, fatty acid storage, and secretion of adipokines and inflammatory mediators. Its composition of smaller adipocytes, stromovascular structures, and immune cells demonstrates distinct metabolic activity. EAT metabolomics is the profiling of metabolites within EAT for identifying pathways and molecular signatures linked to cardiac remodeling and disease progression. Due to its proximity, EAT may directly influence myocardial structure, function, and valvular pathology, making it a potential biomarker and therapeutic target in paradoxical LGAS (low-gradient aortic stenosis)^[2]^.

In patients with aortic stenosis and preserved ejection fraction (ASpEF), EAT thickness is significantly associated with metabolic and inflammatory changes. Thirty-eight metabolites are positively related and 19 are negatively related to EAT at a false discovery rate *P* < 0.01^[3]^. An increase in EAT thickness may worsen valve defects by compressing the myocardium and inducing inflammatory changes. EAT-derived cytokines like IL-6, MCP-1, and TNF-α, being pro-inflammatory and pro-atherogenic substances, promote local and systemic inflammation and aortic valve degeneration, which leads to LV remodeling. AS is strongly age-related, with a prevalence of 13% in people older than 75 years. The interplay between EAT, inflammation, and valvular changes is a complex pathophysiological mechanism. The role of EAT in AS warrants further investigation for potential therapeutic targets^[4]^.

However, certain challenges also exist, as most of the patients with ASpEF are asymptomatic or oligosymptomatic at rest. The interaction between EAT and myocardium in AS remains poorly understood, as only 7% of the patients have low flow, low gradient ASpEF. Another challenge lies in the fact that LV hypertrophy in AS is not only caused by pressure overload but also influenced by factors like insulin resistance, obesity, and diabetes, and these factors are related to EAT, suggesting a role in disease progression. The complex relationship between EAT and substances with both positive and negative correlations, like branched-chain amino acids, also poses a challenge in understanding its pathophysiological role^[5]^.

In conclusion, EAT may play a significant role in the pathophysiology of ASpEF, with implications for therapeutic strategies. Further research is needed to determine whether EAT’s role is active or passive and how metabolic abnormalities contribute to disease progression. EAT volume may be a prognostic factor influencing LV remodeling; however, a consensus on its normal ranges is lacking. Reducing EAT volume may delay disease progression and improve intervention timing, and could be a promising approach to mitigate adverse remodeling in patients with aortic valve stenosis. This letter to editor is in compliance with the TITAN guideline^[6]^.

## Data Availability

No data were generated for this manuscript.

## Research registration unique identifying number (UIN)

Name of the registry: N/A. Unique Identifying number or registration ID: N/A. Hyperlink to your specific registration (must be publicly accessible and will be checked): N/A.
